# A Medical Causerie

**Published:** 1916-03-11

**Authors:** 


### A MEDICAL CAUSERIE.

The recommendations of the Retrenchment Committee, presided over by the Chancellor of the Exchequer, are causing some comment in medical circles. One proposal is to reduce the fee for each certificate issued under the Notification of Infectious Diseases Act from half a crown to one shilling. The Committee hardly help their suggestion by the reasons they advance in its support. To say that doctors have now become familiar with the work seems to imply that the filling up of a certificate and a counterfoil is a task beyond the native capacity of the medical practitioner and one only to be accomplished by special training, and this to a profession which claims to be educated is a hard saying. The second reason—namely, that the amount of labour involved in making out the notification form is "very slight indeed"—shows some conflict with the first, and puts the transaction on a purely clerical basis. Surely the anxiety and responsibility which the diagnosis of a case of infectious disease involves merit some consideration, and if this is worthy of recognition the modest half-crown can hardly be considered excessive. And to say that there are now more diseases to be notified means in substance that as the work is increased the payment ought to be diminished. A frank confession that during the war the country cannot afford notification would have been more to the point. A suggestion has been made in the lay Press that the doctors should volunteer to give up their notification fees until peace is re-established, and possibly they will be ready to do so when all other official payments are reduced in the same proportion; or, perhaps, following an illustrious precedent, they might be willing to accept a proportion of the sum due in Exchequer Bonds.

---

The Scottish universities and colleges get a large share of the new Royal Society Honours. Among the fifteen candidates selected by the Council of the Society for the much-coveted F.R.S. are the names of Dr. J. A. MacWilliam, Professor of Physiology in the University of Aberdeen; Mr. D'Arcy W. Thompson, C.B., Professor of Natural History, University College, Dundee; and Mr. Geo. Gerald Henderson, Professor of Chemistry, Glasgow Technical College. Professor MacWilliam's name is associated with many investigations which have had practical value in medicine, and in recent years he has paid much attention to the sphygmometer and to the clinical estimation of blood pressure. Professor D'Arcy Thompson's original work has been concerned mainly with the scientific relations of pisciculture, and he represented the British Government at the Behring Sea Fisheries Conference as well as on other occasions; he has also cultivated the classical aspect of the subject which he professes, as is seen in his "Glossary of Greek Birds." By Glasgow medical students of the 'nineties Professor Gerald Henderson will be remembered as University Assistant to the Chair of Chemistry. For the last twenty years or so he has been Professor of Chemistry in the Technical College, Glasgow, and has won for himself a high reputation both as an original worker and as a teacher. As the Chair of Chemistry in the University is now vacant by the resignation of Professor Ferguson, it is quite on the cards that Dr. Henderson may return to the teaching staff of his *Alma Mater*. His former University pupils will certainly be disappointed if this anticipation is not justified by the event.

---

Sir John Bland-Sutton recently told a story of one of his house surgeons, who, when asked which he considered the more painful, a chapped lip or a fissured anus, answered, "I don't know, for at the present moment I've got both." The story has a distinctly disappointing quality. Here was a young man in an exceptionally favourable position to settle a question of much interest, and yet he proved unequal to the occasion, and it may be long before such a chance repeats itself. In the meanwhile the issue remains wrapped in uncertainty, which would hardly have been the case had the experience happened to the chief instead of to the assistant. In such an atmosphere one would have expected a greater passion for exact observation. It is sad to think that the chance may never recur. A similar disaster, at least in appearance, once befell Lord Kelvin. He had asked a student in his class "What is electricity?"—and the nervous and hesitating youth produced the apologetic answer "I really did know sir, but at the moment I have forgotten." Throwing up his hands in a gesture of despair, the Professor remarked, "Gentlemen, a great intellectual disaster comparable to the burning of the library at Alexandria has befallen the human race. Here, in our midst, we have a gentleman who once really knew what electricity is, and—he has forgotten!"

---

As an indirect outcome of the economy campaign there is a note by a Harley Street physician in the current number of the *British Medical Journal* drawing attention to the multiple clinical values of the urine test glass. It is reported to be an admirable stethoscope, a suitable apparatus for heavy percussion and for detection of the tendon jerks and the plantar response, and of value in distinguishing pigmented and hæmorrhagic spots in the skin from those due to inflammation—all these in addition to the special purpose for which it was called into being. In these days of costly medical and surgical contrivances it is refreshing to find that it is still possible to combine efficiency with the simple clinical life. A surgeon, famous in his day as an operator, used to boast that he would undertake any operation with the contents of his pocket-case. But he has been dead for many years.

